# Structural and Functional Analysis of the Lectin-like Protein Llp1 Secreted by *Ustilago maydis* upon Infection of Maize

**DOI:** 10.3390/jof11020164

**Published:** 2025-02-19

**Authors:** Marvin Christ, Itzel Rubio Elizalde, Paul Weiland, Antonia Kern, Thomas Iwen, Christopher-Nils Mais, Jan Pané-Farré, Stephan Kiontke, Florian Altegoer, Johannes Freitag, Gert Bange

**Affiliations:** 1 Center for Synthetic Microbiology (SYNMIKRO), Departments of Biology and Chemistry, University of Marburg, Karl-von-Frisch Straße 14, 35043 Marburg, Germany; 2 Institute of Microbiology, Heinrich-Heine University, Universitätsstraße 1, 40225 Düsseldorf, Germany; 3 Max-Planck-Insitute for Terrestrial Microbiology, Karl-von-Frisch Straße 14, 35043 Marburg, Germany

**Keywords:** *Ustilago maydis*, lectin, cell wall, biotrophy, redundancy, smut fungi

## Abstract

The biotrophic fungus *Ustilago maydis*, which causes smut disease in maize, secretes numerous proteins upon plant colonization. Some of them, termed effectors, help to evade plant defenses and manipulate cellular processes within the host. The function of many proteins specifically secreted during infection remains elusive. In this study, we biochemically characterized one such protein, UMAG_00027, that is highly expressed during plant infection. We show that UMAG_00027 is a secreted protein with a lectin-like fold and therefore term it Llp1 (lectin-like-protein 1). Llp1 decorated the fungal cell wall of cells grown in axenic culture or proliferating in planta, which is in agreement with its potential sugar-binding ability. We were unable to identify the precise sugar moieties that are bound by Llp1. CRISPR/Cas9-mediated deletion of *llp1* reveals that the gene is not essential for fungal virulence. A structural search shows the presence of several other lectin-like proteins in *U. maydis* that might compensate for the function of Llp1 in ∆*llp1* mutants. We therefore speculate that Llp1 is part of a family of lectin-like proteins with redundant functions.

## 1. Introduction

*Ustilago maydis* is a fungal pathogen that infects maize, leading to the formation of tumor-like galls known as “corn smut” on the plant’s ears, leaves, and stalks. It is a widely studied model organism for uncovering the molecular mechanisms of plant–pathogen interactions and the colonization of host plants [1,2]. Plant infection by the biotrophic basidiomycete *U. maydis* is achieved after mating of compatible strains. It involves the formation of dikaryotic hyphae and of appressoria-like structures that develop upon contact with the hydrophobic plant surface at hyphal tips to invade the host tissue [3].

To colonialize the host plant, *U. maydis* secretes diverse effector proteins, many encoded in gene clusters [4]. These effectors can counteract plant defenses and manipulate the host cell metabolism. For instance, Pep1 and Tin2 inhibit key apoplastic enzymes, a peroxidase and protease, respectively [5,6,7,8,9]. Tin2 also boosts anthocyanin biosynthesis in maize while reducing lignin, aiding *U. maydis* proliferation [8]. Additionally, the secreted chorismate mutase Cmu1 converts chorismate to prephenate, lowering salicylic acid precursor levels and weakening plant immunity [10,11]. However, Cmu1 is neutralized by maize kiwellin proteins (Kwl1a and Kwl1b), showcasing an evolutionary arms race [12,13,14].

During infection, pathogens rely not only on proteins translocated into the apoplast or host cells but also on the critical roles of cell wall-binding proteins. These proteins can help adhesion to the surface, maintain structural integrity, and hide the fungal cell wall to evade recognition by the host [15]. The fungal cell is surrounded by a complex cell wall composed of interwoven polysaccharides and proteins. The inner layer features a highly cross-linked chitin–glucan matrix, while the outer layer is rich in mannosylated proteins. Chitin is a polymer of β-1,4-linked N-acetylglucosamine, and the glucan matrix consists of β-1,3- and β-1,6-glucan chains [16]. Fungal pathogens are detected by plants through conserved microbe-associated molecular patterns (MAMPs) like chitin and β-glucans [17], which are recognized by the plant’s immune system after being solubilized by host enzymes [18]. This triggers pattern-triggered immunity (PTI), leading to the production of antimicrobial compounds and defense-related proteins [19]. To evade this defense, fungi secrete effectors such as LysM and β-glucan-binding proteins to neutralize these MAMPs and suppress PTI [20,21].

**Figure 1:**
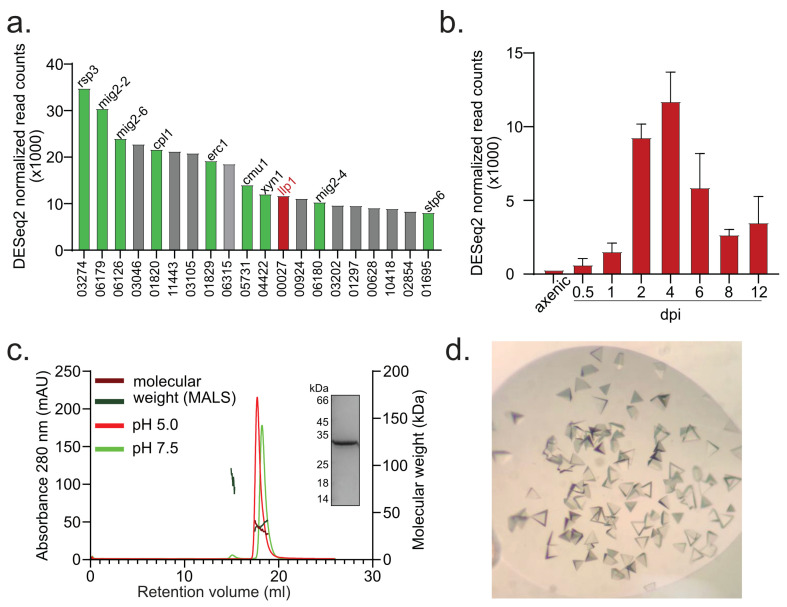
Llp1 is one of the highest upregulated transcripts during the early infection phase and persists in a monomeric conformation and crystallized in three-dimensional pyramids. (**a**) Top 20 abundant transcripts with their respective UMAG-number during *U. maydis* infection at 4 days post-infection (dpi). Characterized effector proteins are shown in green and uncharacterized in gray. Llp1 is marked in red. (**b**) Number of Llp1 transcripts under axenic conditions and during infection. (**c**) Size-exclusion coupled with multi-angle light scattering (SEC-MALS) analysis of Llp1 reveals a predominantly monomeric organization at both pH 7.5 (green) and pH 5.0 (red). At pH 7.5, a minor fraction of the protein remains in a tetrameric state. The inset shows a Coomassie-stained SDS-PAGE gel of the peak fraction (**d**) Three-sided pyramidal crystals of Llp1 appeared within 7 days.

Recently we have characterized an effector protein from *U. maydis* termed Cpl1 (cerato-platanin-like protein 1) [22]. Cpl1 is localized on the fungal cell wall due to its chitin-binding abilities. Deletion of *cpl1* in *U. maydis* mildly affects virulence; however, deletion of its structural homolog Uvi2 (*Ustilago hordei* virulence factor 2) in the closely related smut fungus *Ustilago hordei* strongly interferes with the pathogenic development [23]. In mass-spectrometry-coupled immunoprecipitation experiments using Cpl1 as bait, several cell wall-degrading and decorating proteins were identified, indicating a role of Cpl1 for cell wall modification. Members of this network include the protein disulfide isomerase Pdi1 (UMAG_10156) [24], the endoglucanase Egl1 (UMAG_06332) [25], the endo-1,4-β-xylanase (UMAG_04422) [26], the 1,3-β-glucanase Erc1 (UMAG_01829) [27], and Rsp3, a mannose-binding protein with a shielding function against two antifungal proteins of *Zea mays* [28]. One of the uncharacterized proteins from this cell wall decorating protein network is Llp1 (UMAG_00027). Its transcript belongs to the most abundant transcripts of *U. maydis* at 4 days post-infection (dpi) (Figure 1a,b) [29].

Here, we characterized UMAG_00027 through structural and functional analyses, revealing that this protein has a lectin-like fold and is associated with the fungal cell wall. Thus, we suggest the name “Lectin-like protein 1” (Llp1). Although its expression is significantly upregulated during the biotrophic phase, deletion of *umag_00027* did not interfere with virulence. We detected further proteins with a similar fold suggesting a redundant network of lectin-like-proteins that control the organization of the fungal cell wall upon plant infection by *U. maydis*.

## 2. Materials and Methods

### 2.1. Protein Production and Purification

The gene *umag_00027* encoding full-length Llp1 (Uniprot ID: A0A0D1E6R1) was obtained as synthetic DNA (IDT) optimized for expression in *Escherichia coli*. For recombinant protein production, a fragment containing amino acid residues 23–241 of Llp1, lacking the signal peptide, was amplified using an Expand High Fidelity PCR system (Roche, Indianapolis, IN, USA) and inserted into a pET-24d vector (Novagen, St. Louis, MO, USA) at customized BsaI (NEB) restriction sites [30]. Constructs included a hexahistidine (His_6_) tag at the C-terminus for purification. All constructs were verified by sequencing. Plasmids and primers utilized in this study are listed in Supplementary Tables S1 and S2.

The Llp1 protein was expressed in *E. coli* SHuffle T7 (Novagen, St. Louis, MO, USA), cultured in lysogeny broth (LB) medium supplemented with 1.0% (*w*/*v*) galactose for 16 h at 30 °C. To produce selenomethionine-labeled Llp1, *E. coli* SHuffle T7 cells were transformed with the pET24d-*llp1* plasmid, grown on LB agar with 50 μg/mL kanamycin at 37 °C for 16 h, and used to start a preculture. This was then inoculated into 5 L of M9 medium (37.25 g/L Na_2_HPO_4_, 16.5 g/L KH_2_PO_4_, 2.75 g/L NaCl, 5.5 g/L NH_4_Cl, pH 7.5) supplemented with 50 μg/mL kanamycin and SolX supplements to an OD_600_ of 0.1. The composition of the SolX solution was: 1 g/L l-lysine, 1 g/L l-threonine, 1 g/L l-phenylalanine, 0.5 g/L l-leucine, 0.5 g/L l-isoleucine, 0.5 g/L valine, 0.25 g/L selenomethionine, 80 g/L glucose, 100 mM MgCl_2_, and 10 mM CaCl_2_. After growing to an OD_600_ of 0.6 at 37 °C, protein production was induced with 1 mM IPTG, followed by incubation for 24 h.

Cells were collected by centrifuging at 4000× *g* for 15 min at 10 °C. The resulting cell pellet were resuspended in 10 mL of lysis buffer per gram of cells and processed using an M-110L Microfluidizer (Microfluidics, Westwood, MA, USA). The lysis buffer was composed of 20 mM HEPES pH 8.0, 20 mM KCl, 20 mM MgCl_2_, 40 mM imidazole, 250 mM NaCl. After lysis, the mixture was clarified through centrifugation at 50,000× *g* for 30 min at 5 °C using a A27-8 x 50 rotor (ThermoFisher Scientific, Waltham, MA, USA). The cleared lysate was then applied to a 1 mL HisTrap HP column (GE Healthcare, Chicago, IL, USA), which was initially washed with 10 column volumes of lysis buffer. The protein was eluted using lysis buffer (pH 8.0) supplemented with 250 mM imidazole. The eluate was then concentrated to about 30 mg/mL using an Amicon Ultracel-10K (Millipore, Burlington, MA, USA) and further purified by size-exclusion chromatography on a Superdex 200 XK 26/600 column (GE Healthcare, Chicago, IL, USA) with the same buffer (pH 7.5), excluding imidazole. Protein-containing fractions were pooled and concentrated to the required final concentrations.

### 2.2. Protein Crystallization and Structure Determination

Crystallization was carried out using the sitting-drop method at 20 °C with drop sizes of 0.5 μL. The drops contained a mixture of protein and precipitant solutions at ratios of either 1:1 or 1:2. Crystallization drops were prepared automatically using a Crystal Gryphon robot (Art Robbins Instruments, Sunnyvale, CA, USA). The NeXtal JCSG suites I–IV were used to screen for optimal crystallization conditions. Native Llp1 crystals formed at a concentration of 0.6 mM within 7 days in various solutions. Selenomethionine-labeled Llp1 crystallized at 0.6 mM within 14 days under similar conditions. Crystals were flash-frozen in liquid nitrogen with cryo solution composed of the crystallization solution supplemented with 20% vol/vol glycerol and stored constantly in liquid nitrogen conditions.

Data were collected under cryogenic conditions at the EMBL P14 beamline (Deutsches Elektronen-Synchrotron; DESY, Hamburg, Germany) and processed using XDS and XSCALE [31]. The structure of Llp1 was solved by isomorphous replacement, utilizing single-wavelength anomalous dispersion data obtained from selenomethionine incorporation. COOT [32] was used to manually build and PHENIX [33] to refine the structure. Graphical presentation of the structure was performed with ChimeraX [34].

### 2.3. Size-Exclusion Chromatography-Multi-Angle Light Scattering

SEC-MALS was performed using an ÄKTA PURE system (GE Healthcare, Chicago, IL, USA) equipped with a Superdex 200 Increase 10/300 column, a MALS detector 3609 (Postnova Analytics, Landsberg am Lech, Germany), and a refractive index detector 3150 (Postnova Analytics). The column was equilibrated with HEPES buffer for analyses at pH 7.5 or citrate buffer for studies at pH 5.0. Molecular weights were calculated by combining refractive index and MALS data using Zimmermann fitting.

### 2.4. Generation of U. maydis Strains

*U. maydis* protoplasts were generated and transformed as described previously [35,36]. Briefly, 10 µL of donor DNA (100 ng/µL) and 500–1000 ng of plasmid-DNA in a maximum volume of 10 µL were added together with 1 µL heparin (15 mg/mL) to the protoplasts and were incubated on ice for 10 min. Subsequently, 500 μL of ice-cold sterile STC solution (1 M sorbitol, 10 mM Tris–HCl pH 7.5, 100 mM CaCl₂) supplemented with 40% (*w*/*v*) polyethylene glycol (PEG) 3350 was added, and the mixture was incubated for an additional 15 min on ice. The total volume was plated onto double-layered regeneration agar plates (10 g/L yeast extract, 20 g/L Bacto-peptone, 20 g/L sucrose, 1 M sorbitol, 15 g/L agar): the bottom layer was supplemented with 4 μg/mL carboxin, and the top layer consisted of regeneration agar without antibiotics. After 4–7 days of incubation at 28 °C, transformants were chosen and analyzed for successful transformation. Disruption of the *llp1* gene in the respective *U. maydis* strain was conducted by using the CRISPR/Cas9 approach for genetic manipulation as described previously [37]. Donor DNA with 40 nucleotides overhang of the left and right border of the target gene was supplied during the transformation to delete the open reading frame without affecting neighboring genes. Successful *U. maydis* transformants were confirmed using colony PCR with specific primers (Supplementary Figure S1). For generating knockouts, the plasmid pMS73 was digested with Acc65I (New England Biolabs, Ipswich, MA, USA), and the respective single guide RNA was integrated using Gibson assembly, according to the protocol described previously [37]. The target sequence was designed using an E-CRISP tool (Supplementary Table S1) [38]. The construction of the HA-tagged *llp1* followed the same procedure as the knockout of the gene, except that the donor DNA encoding an HA tag with flanking sequences was designed for the C-terminus of Llp1. All plasmids were validated by sequencing.

### 2.5. Immunolocalization on an Artificial Surface and in Planta

For localization of Llp1-HA in filamentous hyphae, the *U. maydis* strain constitutively expressing Llp1-HA was grown to an OD_600_ of 0.8 in YEPSlight and resuspended in 2% YEPSL containing 0.1 mM 16-hydroxy hexadecanoic acid (Sigma, St. Louis, MO, USA) to a final OD_600_ of 0.6 and 2 mL cell suspensions sprayed onto Parafilm M (Carl Roth, Karlsruhe, Germany). The Parafilm M was placed on wetted paper inside square petri dishes to ensure adequate humidity and incubated at 28 °C for 16 to 18 h. Following incubation, the Parafilm M was washed with PBS (40 mM KH_2_PO_4_, 160 mM Na_2_HPO_4_, 1.1 M NaCl, 0.1% Tween-20, and pH 7.4), blocked with 3% (*w*/*v*) BSA, and incubated overnight at 4 °C in PBS containing an α-HA antibody (Sigma; 1:1500 dilution) and 3% (*w*/*v*) BSA. The next day, after washing with PBS, the samples were incubated for 1 h at room temperature with a goat anti-rabbit IgG secondary antibody conjugated with Alexa Fluor 488 (Life Technologies, Carlsbad, CA, USA; 1:1500 dilution). After washing, the samples were analyzed using a confocal laser fluorescence microscope (SP8, Leica, Bensheim, Germany).

For immunostaining Llp1-HA in biotrophic hyphae, infected leaves were harvested at 4 days post-infection, and the epidermal layers were peeled off. Leaves were treated with cell wall macerating solution (10 mM MES pH 5.7, 1.5% (*w*/*v*) cellulase from *Trichoderma* sp. (Serva), 0.3% macerozyme R10 (Serva), 0.6 M mannitol, 1 mM CaCl₂, and 0.1% (*w*/*v*) BSA) for 90 min, then washed with PBS and fixed in 4% (*v*/*v*) paraformaldehyde for 30 min at room temperature. After washing with PBS, the leaves were incubated with 0.1 M glycine in PBS for 15 min. Antibody incubation and microscope procedure followed the same protocol used for hyphae on Parafilm M.

### 2.6. MicroScale Thermophoresis

MicroScale Thermophoresis (MST) experiments were conducted in HEPES buffer with 0.05% (*v*/*v*) Tween 20, using a Monolith NT.115 instrument (NanoTemper Technologies, Munich, Germany). The Excitation-Power (red LED) was set to 5–10% and the infrared laser power to 75% for each of the three replicates. The Llp1 protein was labeled following the supplier’s protocol (dye NT 647, NanoTemper Technologies, Munich, Germany). A fixed concentration of 500 nM of labeled protein (200 nM in the case of chitobiose) was titrated against decreasing concentrations of sugars, in the range of 10 mM to 152 nM. To test binding of calcium and manganese ions, a range of 2.5 mM to 76 nM was used. MST data were recorded at 680 nm, using premium capillaries (NanoTemper Technologies, Munich, Germany), and analyzed using MO. Affinity Analysis v2.3. The results are summarized in Supplementary Table S4.

### 2.7. Zea mays Infection Assay

The corresponding *U. maydis* strain was grown in YEPSlight medium to a final OD_600_ of 0.8. The cultures were then adjusted to an OD_600_ of 1 using autoclaved double-distilled water. A measure of 500 μL of each culture was injected into the stems of 7-day-old maize seedlings using a syringe, following the method described by Kämper and colleagues [4]. For FB1 and FB2, cultures were mixed in a 1:1 ratio before injection. The disease symptoms of the infected plants were evaluated over a period of 12 days post-infection and quantified based on three biological replicates within a total of more than 100 analyzed plants. The results are summarized in Supplementary Tables S5 and S6.

### 2.8. Fungal Stress—And Filamentation Assay

Fungal strains were cultured in YEPSlight until reaching an OD_600_ of 1.0. The cells were then pelleted and resuspended in autoclaved double-distilled water to an OD_600_ of 1. For the stress assay, the corresponding strain was then spotted with 10 µL in serial 10-fold dilutions on CM plates [39] supplemented with the different stressors with the following concentrations: 150 µg/mL Congo Red, 200 µg/mL Calcofluor White, 1.5 mM H_2_O_2_, 1 M NaCl, 0.003% sodium-dodecyl-sulfate (SDS). For the filamentation assay, PDA plates containing 1% charcoal were used [40]. FB1 and FB2 strains were spotted with 10 µL of their respective mating partners in a grid pattern, or alone, while SG200 was spotted in 10 µL serial 10-fold dilutions. Close-up images of filaments were captured at 40× magnification.

### 2.9. Protein Production with Ustilago maydis and Immunoblotting

Protein production in U. maydis was conducted as previously described [41]. Briefly, fungal strains expressing C-terminally HA tagged Llp1 from its native locus were cultivated overnight in YEPSlight medium. The following day, the overnight culture was used to inoculate 50 mL of fresh medium to an OD_600_ of 0.1. Cultures were supplemented with 1.5% glucose and incubated at 28 °C for 72 h under constant shaking at 180 rpm. Following cultivation, 1.5 mL of culture was centrifuged at 18,800× *g* for 1 min to separate cells from the supernatant. The cell pellet was resuspended in 40 µL of 0.5 M NaOH, and 10 µL of 5× Laemmli buffer was added. For the supernatant fraction, 375 µL of 100% trichloroacetic acid (TCA) was added, and the sample was inverted five times. After incubation at 4 °C for 10 min, precipitated proteins were pelleted by centrifugation at 18,800× *g* (4 °C). The supernatant was carefully removed, and the pellet was washed with 200 µL of ice-cold acetone. The washing step was repeated twice. After drying the pellet at 95 °C for 10 min, it was resuspended in 40 µL of lysis buffer (excluding imidazole, see Section 2.1), and 10 µL of 5× Laemmli buffer was added. Both pellet and supernatant samples were incubated for 10 min at 95 °C before loading an SDS-PAGE for analysis.

For immunoblotting, proteins separated by SDS-PAGE were transferred onto a PVDF membrane using a Trans-Blot Turbo Transfer System (Bio-Rad, Hercules, CA, USA), following the manufacturer’s protocol. The membrane was then blocked for 1 h at room temperature in PBS buffer (Section 2.5) supplemented with 10% skimmed milk powder. For immunodetection, the membrane was incubated in 10 mL of PBS containing the α-HA antibody (Sigma, produced in rabbit; 1:10,000 dilution) for 1 h at 10 °C. Following three washes with PBS, the membrane was incubated in 10 mL of PBS containing an HRP-conjugated secondary antibody (Cell Signaling Technologies; 1:10,000 dilution) specific to the primary antibody, for 1 h at room temperature. After additional washes, chemiluminescent signals were generated (Cytiva, Marlborough, MA, USA) and analyzed using an Azure Biosystems imaging system (Dublin, CA, USA).

## 3. Results

### 3.1. Structural Analysis of Llp1 Reveals a Lectin-like Protein

The gene *UMAG_00027* encodes for the 249-amino-acid protein Llp1 with an estimated molecular weight of 25 kilo Dalton (kDa), exclusively found in smut fungi (Figure 1c; Supplementary Figure S2a). In silico analysis by SignalP [42] and CCTOP [43] software revealed an N-terminal signal peptide of 22 amino acids and no transmembrane helices. Except for *Sporisorium graminicola*, all the identified orthologs bear an N-terminal signal peptide. In addition, all of them contain six conserved cysteine residues (Supplementary Figure S2b). During the infection of *Ustilago maydis*, *llp1* is one of the most highly expressed genes at day 4 post-infection, reaching its peak of mRNA counts of almost 12,000 (Figure 1a,b).

To gain deeper insight into Llp1, we sought to solve its crystal structure. The protein fused to a C-terminal hexa-histidine tag, was expressed in *Escherichia coli* SHuffle T7 cells and enriched via a two-step purification protocol combining nickel ion affinity chromatography and size exclusion chromatography (SEC). This process yielded a protein with high purity, as confirmed by Coomassie-stained SDS-PAGE, and predominantly monomeric organization as determined by SEC-MALS analysis. (Figure 1c).

**Figure 2:**
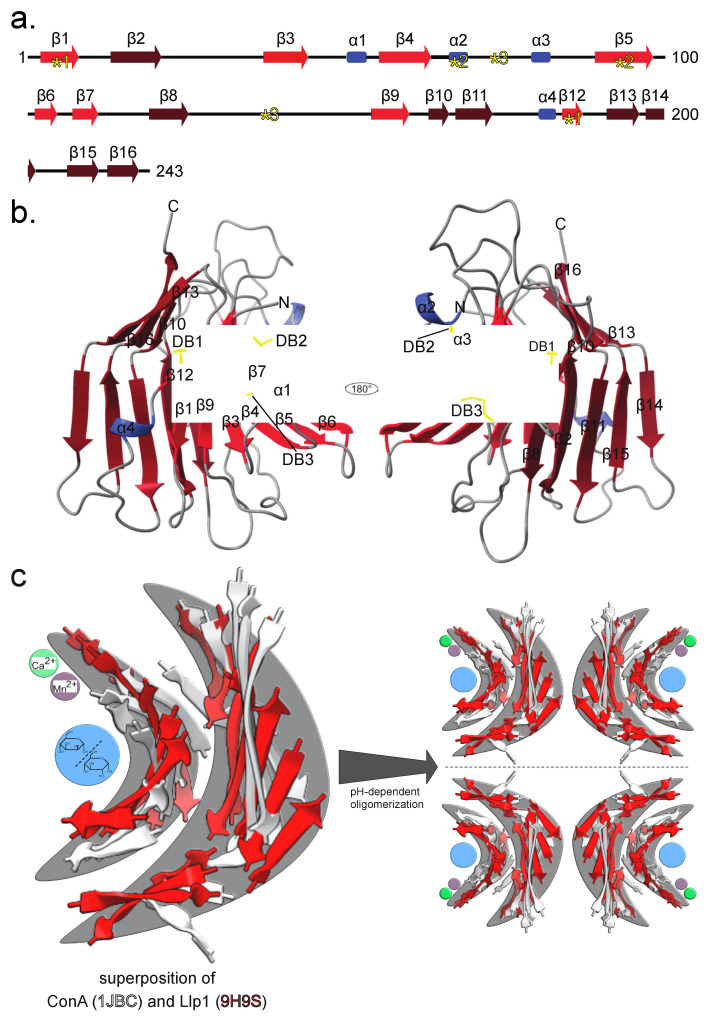
Llp1 structure reveals a β-sandwich with a lectin-like structure. (**a**) Secondary structure organization of the Llp1. The yellow, numbered asterisks indicate the position of the six cysteines that form disulfide bridges 1 to 3. (**b**) Cartoon model of Llp1 colored by secondary structure elements in red and blue and disulfide bridges in yellow. (**c**) Superposition of the β-sandwich ribbon of Llp1 (red) and ConA (white, 1JBC). The characteristic concave and convex architecture of the β-strands is highlighted in gray. The metal binding sides are shown in purple and green. The blue sphere is intended to represent the sugar-binding pocket. Shown are glucose and mannose molecules.

Initial crystals of Llp1 were grown in sitting-drop vapor-diffusion experiments, where the reservoir solution consisted of 0.1 M HEPES pH 7.5 and 1.26 M ammonium sulfate (D2; NeXtal JCSG Core Suite IV). Three-sided pyramidal crystals appeared within 7 days (Figure 1d). Diffraction data were collected at 2.01 Å resolution at the EMBL beamline P14 (Deutsches Elektronen-Synchrotron; DESY) under cryogenic conditions. Due to the absence of known structures suitable for molecular replacement, we employed isomorphous replacement using single-wavelength anomalous dispersion data obtained from selenomethionine incorporation. Subsequently, the structure was manually constructed in Coot [32], and refined to R*_work_* and R*_free_* values of 0.19 and 0.22, respectively (Table 1). Amino acid residues 23–241 of Llp1 could be unambiguously assigned to the electron-density map and represent almost all the residues of the protein construct employed in this study (Note: N-terminal 22 residues comprising the signal peptide were not present in the Llp1 expression construct). The structure of Llp1 (PDB: 9H9S) reveals that the protein is predominantly an all-β protein, composed of a β-sandwich formed by two eight-stranded antiparallel β-strands (Figure 2). These β-strands are arranged into two major antiparallel segments, creating a concave upper and a convex lower surface. The secondary structure elements are connected by several loops, which contain four small helices. In total, the molecule has three exposed disulfide bonds probably conserved among orthologs (Figure 2a,b; Supplementary Figure S2b). Two of the disulfide bonds are located in the upper sheet. One connects the small β-strand 12 to β-strand 1 (Figure 2a). The other disulfide bond links the small α-helix 2 in the long loop to β-strand 5. The third disulfide bond keeps the connecting loop between β8 and β9 close to the molecule. Overall, this results in a compact and stable molecular fold. Taken together, our structural analysis shows that Llp1 has a high structural similarity to members of the carbohydrate-binding concanavalin A-like (ConA) lectin/glucanase family [44]. The superposition of the characteristic double β-sheets, excluding the connectors, loops, and helices, revealed a root mean square deviation (RMSD) of the Cα-atoms of 6.845 Å over 103 of 106 Cα-atoms (Figure 2c). The sequence identity is 25.98% and the similarity 40.21%. ConA lectins, named after the first lectin, that was structurally characterized from *Canavalia ensiformis*, are present in all kingdoms of life [45]. Proteins that share this fold have a high variety of functions, ranging from inflammatory and antitumor activities to acting as antibacterial as well as antifungal agents [46]. Biochemically, ConA lectins have three features (Figure 2c): binding of divalent metals like calcium and/or manganese, recognizing and binding of sugars without hydrolyzing the carbohydrate, and the ability to build pH-dependent oligomers. The binding of sugars in this type of molecule is limited to glucose and mannose and their mono- or disaccharide derivatives. The binding of trisaccharides was only observed in the case of trimannosides [44].

### 3.2. Llp1 Localizes at the Surface of Fungal Hyphae—But Not Plant Cells During Infection

Our structural analysis revealed that Llp1 has a lectin-like structure, suggesting that the protein may have the capacity to bind sugars as well as metals. Despite extensive efforts with microscale thermophoresis (MST), we could not detect the specific binding of Llp1 to a variety of sugars such as chitobiose, chitotetraose, N-acetylglucosamine, rhamnose, cellobiose, galactose, arabinose, mannose, maltotriose, and glucose. Moreover, no binding to typical ligands such as calcium and manganese was detected (Supplementary Table S4). A notable feature of some lectin-like proteins is their pH-dependent oligomerization. During SEC-MALS experiments conducted at both neutral and acidic pH, the protein predominantly displayed a monomeric organization. The theoretical mass of a monomer is 25.8 kDa. For the two peaks representing the monomeric form, calculated masses of 30.2 kDa (±6%) and 35.9 kDa (±4.7%) were observed at pH 7.5 and pH 5.0, respectively. Additionally, a small secondary peak at pH 7.5 corresponded to a measured mass of 83.6 kDa (±10.9%), indicating its potential to adopt a trimeric conformation (Figure 1c).

**Figure 3:**
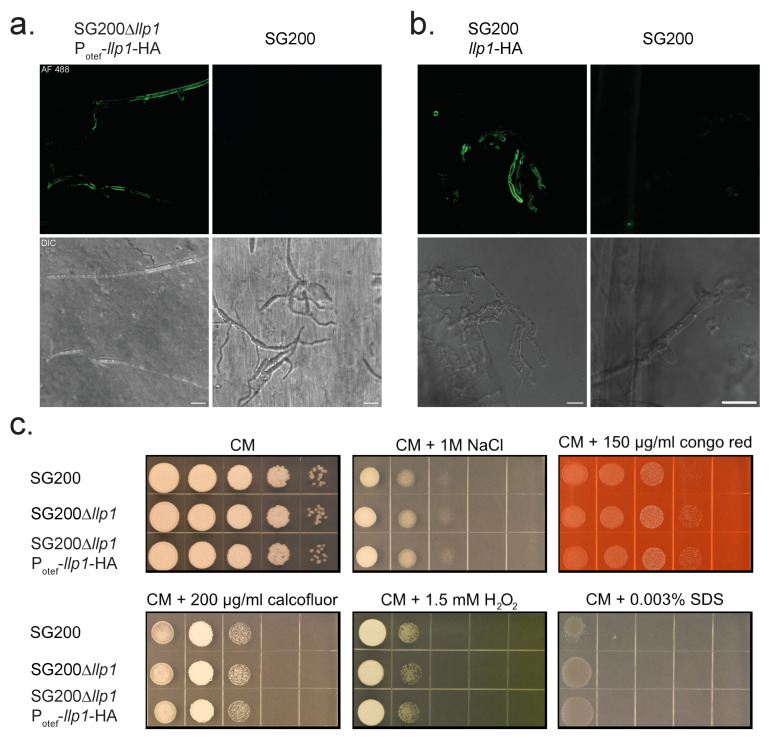
Llp1 is masking the fungal but not the plant cell wall, and it appears not to affect cell wall or membrane integrity or responses to osmotic stressors. (**a**) HA-tagged *llp1* under the control of the constitutive *otef* promoter complementing a SG200 *llp1* deletion strain is secreted and decorates the fungal cell wall of *U. maydis* hyphae grown on a Parafilm M surface. (**b**) *llp1* is HA-tagged in its native locus and is secreted during infection decorating only the fungal but not the plant cell wall. (**a**,**b**) Confocal microscopy of respectively SG200 strains expressing HA-tagged *llp1* (left) and SG200 without HA-tagged gene (right) incubated with primary HA-tag antibody and AF488 conjugated secondary antibody. Top panels show images with green fluorescent protein channels and low panels show differential interference contrast images. Scale bar = 10 µm. (**c**) Stress assay of *U. maydis* strains SG200, SG200∆*llp1*, and SG200∆*llp1*-P_OTEF_-*llp1*. Serial 10-fold dilutions, starting with OD_600_ of 1, were spotted on CM agar supplemented with glucose as a carbon source and the respective stressors.

Next, we aimed to conduct an in vivo analysis of the cell wall-binding ability of Llp1. To this end, we created a strain constitutively expressing Llp1 tagged with a hemagglutinin (HA)-epitope. To induce filamentation, we incubated this strain with hydroxy-fatty acids on Parafilm M to mimic the hydrophobic surface of the plant [47]. Wildtype SG200 cells served as the control. Llp1-HA was detected with anti-HA antibody and an AF488-conjugated secondary antibody in fixed and permeabilized cells. We observed a fluorescence decorating the exterior of *U. maydis* hyphae in the strain expressing Llp1-HA and no fluorescence in the control strain (Figure 3a). The absence of fluorescence in the areas surrounding the filaments reveals that Llp1-HA is closely associated with the fungal cell wall.

To assess whether Llp1, expressed under control of its native promoter, attaches to fungal hyphae during colonization, leaf samples infected with SG200 Llp1-HA were harvested 4 dpi and were partially macerated and then subjected to anti-HA immunostaining as described before [28,47]. Tight association of Llp1 to the fungal cell wall was confirmed in this experiment (Figure 3b). Decoration of plant tissue could not be detected. These results show that Llp1 specifically binds to fungal—but not the plant—cells during *U. maydis* infection. When the protein was produced in *U. maydis* axenic cultures as described before [41], and the culture was separated into a cell pellet and a supernatant fraction by centrifugation, a significant amount of secreted, HA-tagged Llp1 remained detectable in the cell pellet fraction via immunoblotting (Supplementary Figure S3), corroborating its interaction with the cell wall. While efforts no specific sugar binding to the protein was identified, the association of Llp1 with the cell wall prompted us to investigate whether cell wall stress impacts *U. maydis* strains lacking or overexpressing Llp1. To assess this, we spotted serial dilution of these strains on media containing glucose as the carbon source and various stress elicitors: Congo Red and Calcofluor White (cell wall stress), H₂O₂ (oxidative stress) and NaCl (osmotic stress) and SDS (membrane stress). Under all tested conditions, no relevant effect was observed.

### 3.3. Llp1 Is Obsolete for the Infection by U. maydis and Potential Part of a Redundant Network of Surface Proteins

**Figure 4:**
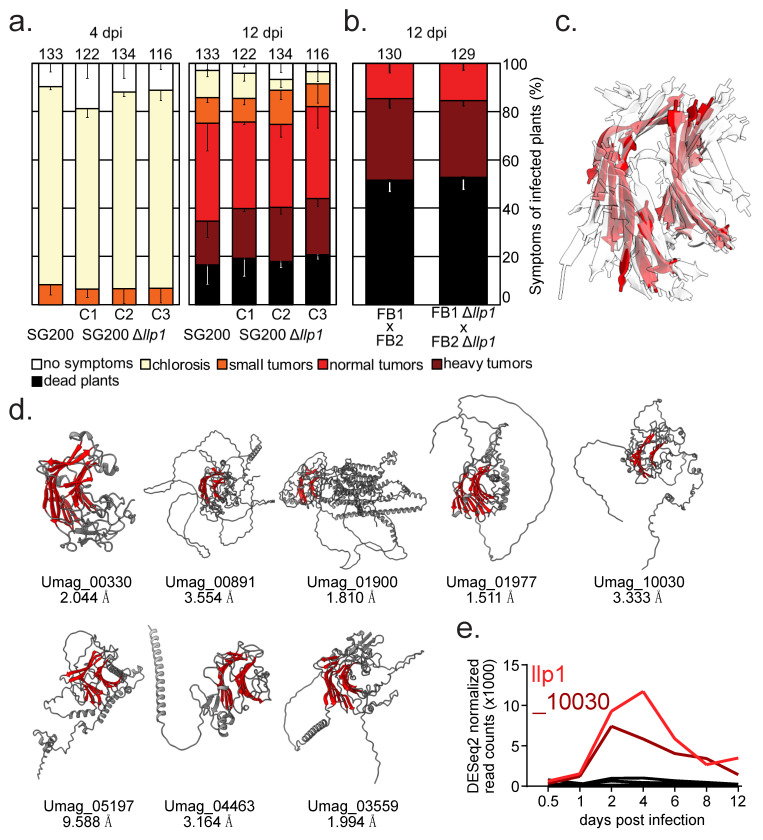
Llp1 is obsolete for the infection and *U. maydis* has more Llps that are regulated during the infection. (**a**) Maize infection assay using deletion strains in the solopathogenic strain SG200 at 4 and 12 dpi (**b**). Maize infection assay using deletion strains in the haploid strains FB1 and FB2 at 12 dpi (**c**). Superposition of the β-sheet core from all identified lectin-like proteins, with Llp1 highlighted in red. (**d**) AlphaFold models of the identified Llp proteins. The β-sheet core of each lectin-like domain is highlighted in red. The number under each gene identifier represents the RMSD value of the Cα superposition of the respective β-sheet core to that of Llp1. (**e**) Transcript levels of all identified lectin-like proteins during *U. maydis* infection of maize.

Finally, we investigated if Llp1 contributes to the virulence of *U. maydis*. Thus, we generated ∆*llp1* deletion strains in the solopathogenic strain SG200, which does not require a mating partner to infect maize by means of the CRISPR/Cas9 system [36,37]. Seven-day-old maize seedlings were infected with three independent transformants lacking *llp1*. The symptoms of *U. maydis* infection were monitored over the whole period of infection (Supplementary Table S5). No effects were observed, either at 4 dpi, when its transcript levels peak, or at 12 dpi (Figure 4a). To initiate tumor formation, haploid strains of compatible mating types fuse to form an infective dikaryotic filament [48]. We reasoned that cell fusion may require Llp1 as it decorates the cell wall. Cell wall-binding proteins as well as secreted proteins play a crucial role during this process [49]. To test mating and pathogenicity, *llp1* was deleted in the compatible *U. maydis* strains FB1 and FB2 [40]. Again, no effect on virulence was detected upon comparing mutant strains with wildtype strains, suggesting that Llp1 is also obsolete for the mating process (Figure 4b, Supplementary Table S6). Also, a mating and filamentation assay on PD agar containing charcoal [40] did not reveal a difference between the wildtype and mutant (Supplementary Figure S4a). Furthermore, a strain constitutively overexpressing Llp1 showed no differences in filament formation (Supplementary Figure S4b).

Functional redundancy among cell wall-binding proteins has been previously observed [15]. We considered the possibility that another protein may compensate for the loss of Llp1. No similar proteins with a high level of amino acid identity were identified. However, it is well established that proteins with a typical ConA-like fold often share structural similarities despite lacking sequence similarity [50]. Therefore, we conducted a structure-based search with Foldseek [51] using Llp1 as a model. This approach uncovered eight additional proteins in *U. maydis* (UMAG_00330, _00891, _01900, _01977, _05197, _04463, _03559, and _10030; Figure 4c,e) that possess the characteristic lectin-like β-sandwich fold. Superposition of the Cα atoms of the respective β-sheet cores revealed strong similarities, with RMSD values below 2 Å for several of the candidates (Figure 4d). In particular, not all of these proteins consist solely of the ConA β-core. For example, UMAG_01900 appears to be a multidomain protein, with the core accounting for only ~20% of the full protein. In other cases, the β-core is extended by unique secondary structure elements, which are specific to each protein. Examining transcript regulation during infection reveals that *llp1* is the most strongly expressed gene among the identified candidates. Notably, only *umag_10030*, which was also found in the mass-spectrometry-coupled immunoprecipitation experiments using Cpl1 as bait [22], exhibits a transcript level, peaking at 2 days post-infection. All other identified genes show lower expression throughout infection, with peaks at 2 and 4 dpi, and a maximum of 1000 transcripts detected.

## 4. Discussion

### 4.1. Llp1 Is Strongly Upregulated During Pathogenic Development, but Its Exact Role Is Unknown

Our study has identified Llp1 as a lectin-like protein localizing to the fungal cell wall. The protein fulfills many criteria for a smut fungus-specific effector protein. First, it is conserved among several species (Supplementary Figure S2b) and upregulated in the related smut fungus *Sporisorium reilianum* ([52]; Supplementary Figure S5). Moreover, it co-purifies with known effector proteins such as, e.g., Rsp3 and Cpl1, making it part of a potential cell wall-regulating protein network.

However, extensive attempts to decipher phenotypic consequences of *llp1* deletion remained unsuccessful. Even stress assays testing strains with overexpressed Llp1-HA did not show an effect. Such overexpression combined with stress assays has proven useful for characterizing the function of the *Piriformospora indica* lectin FGB1 that suppresses glucan-triggered immunity in plants [21,53]. It is possible that the biological role of Llp1 is not directly related to pathogenic development or cell wall stress resistance but may have a role upon contact with other organisms, e.g., bacteria that contain a cell wall made up of a complex sugar network. It becomes more and more evident that pathogenic fungi face various microbial barriers and plant defense responses during the infection process [54]. Alternatively, it might only be required for colonialization of particular organs. Indeed, it was established previously that certain effector proteins are only relevant for infection of specific tissue [55,56].

Together with previous data on the very highly expressed *U. maydis* effectors 4 days post-infection, we reveal that several proteins encoded by the most abundant transcripts during plant colonialization often play no detectable or only a minor role in the infection process (e.g., Mig-cluster [57,58]; Cpl1 [22]; Cmu1 [10]). An interesting topic for future studies is to explore whether these effectors perform functions not assessed by the standard virulence assay but are nonetheless critical in natural environments, or if they are functionally redundant.

### 4.2. A Group of Functionally Redundant Secreted Lectin Proteins?

It is regularly observed that depletion of individual proteins is compensated by another protein able to fulfill a similar function; e.g., in *U. maydis*, a family of highly similar effectors has been described for which parallel depletion of several members is necessary to observe a phenotype [59]. Another example is the effector pair Hum3 and Rsp1. Both of them are repetitive, secreted proteins with similar functions, play a role in the attachment of fungal hyphae to hydrophobic surfaces, and promote aerial growth [60]. A similar scenario could explain our data for Llp1. Based on its structure, we were able to identify eight candidates, of which UMAG_10030 has a similar expression pattern and is also highly expressed (Figure 4e). Since all the lectin-like proteins identified in *U. maydis* show a very different composition with the exception of the lectin fold (Figure 4d), it might also be that all of them carry out more specific functions. To our knowledge, there are no reported cases of critical functions of lectin-like proteins from fungal pathogens for plant infection, although many exist [61,62]. One exception might be a LysM domain containing lectin in *Magnaporthe oryzae*, which is upregulated during early infection and decorates appressoria [63]. Thus, more research is needed to identify potential roles of this diverse family of proteins for plant infection.

## 5. Conclusions

In this study, we identified a group of lectin-like proteins in *Ustilago maydis* and provide an in-depth characterization of one of these lectins, named Llp1. Our findings set the groundwork to further reveal the role of this important group of potential cell wall-binding proteins and aid other researchers in elucidating how the fungal cell wall is adapted to pathogenic development.

## Figures and Tables

**Table 1:** Crystallographic data collection and refinement statistics.

	Llp1 (UMAG_00027)
**Data collection**	
Space group	F 2 3
Cell dimensions	
*a*, *b*, *c* (Å)	159.95 159.95 159.95
α, β, γ (°)	90 90 90
Resolution (Å)	48.23–2.01 (2.082–2.01)
*R*_merge_	0.1436 (2.947)
*I*/σ*I*	19.10 (0.77)
Completeness (%)	99.88 (98.93)
Redundancy	38.4 (22.8)
CC_1/2_	1 (0.357)
**Refinement**	
Resolution (Å)	48.23–2.01
No. of reflections	22,618
*R*_work_/*R*_free_	0.188/0.216
No. of atoms	1790
Protein	1696
Ligand/ion	5
Water	89
*B*-factors	62.32
Protein	62.51
Ligand/ion	72.48
Water	58.25
Ramachandran (%)	
favored	98.62
allowed	1.38
outliers	0.0
R.m.s. deviations	
Bond lengths (Å)	0.016
Bond angles (°)	1.39

Values in parentheses are for highest-resolution shell.

## Data Availability

The original contributions presented in the study are included in the article/Supplementary Material, further inquiries can be directed to the corresponding author.

## Supplementary Materials

The following supporting information can be downloaded at: https://www.mdpi.com/article/10.3390/jof11020164/s1, Figure S1: Validation of *U. maydis* transformants using colony PCR; Figure S2: Llp1 and homologs are specific to smut fungi; Figure S3: Llp1 produced in *U. maydis* associates with cells and is secreted into the medium; Figure S4: Mating - and filamentation assay on PD agar supplemented with charcoal; Figure S5: The homologue of *umag_00027* in *Sporisorium reilianum* (*sr00846.2*) shares a similar transcript regulation pattern and structural similarity. Table S1: Primers used and generated in this study; Table S2: Plasmids used and generated in this study; Table S3: *Ustilago maydis* strains used and generated in this study; Table S4: MST measurements for sugar- and metal-binding interaction experiments; Table S5 and S6: Virulence testing *of U. maydis* strains lacking llp1 were tested in a maize infection assay. References [4,37,40,52,64] are cited in the supplementary materials.
