# Supplementary material for: Structural and Functional Analysis of the Lectin-like Protein Llp1 Secreted by Ustilago maydis upon Infection of Maize
Source: J Fungi (Basel). 2025 Feb 19;11(2):164. doi: 10.3390/jof11020164 (PMC11857070; doi:10.3390/jof11020164)
Supplement: Supplementary file 1 [file jof-11-00164-s001.zip › Supplementary Information.pdf]

Supplementary Information for:

Structural and Functional Analysis of the Lectin-like Protein Llp1 Secreted by Ustilago maydis upon Infection of Maize

Marvin Christ 1, Itzel Rubio Elizalde 1, Paul Weiland 1, Antonia Kern 1, Thomas Iwen 1, Christopher-Nils Mais 1, Jan Pané-Farré 1, Stephan Kiontke 1, Florian Altegoer 2, Johannes Freitag 1 and Gert Bange 1,3,*

1 Center for Synthetic Microbiology (SYNMIKRO), Departments of Biology and Chemistry,

University of Marburg, Karl-von-Frisch Straße 14, 35043 Marburg, Germany

2 Institute of Microbiology, Heinrich-Heine University, Universitätsstraße 1, 40225

Düsseldorf, Germany

3 Max-Planck-Insitute for Terrestrial Microbiology, Karl-von-Frisch Straße 14, 35043

Marburg, Germany

* Correspondence: gert.bange@synmikro.uni-marburg.de

The file contains:
Supplementary Figures S1-S5
Supplementary Tables S1-S3

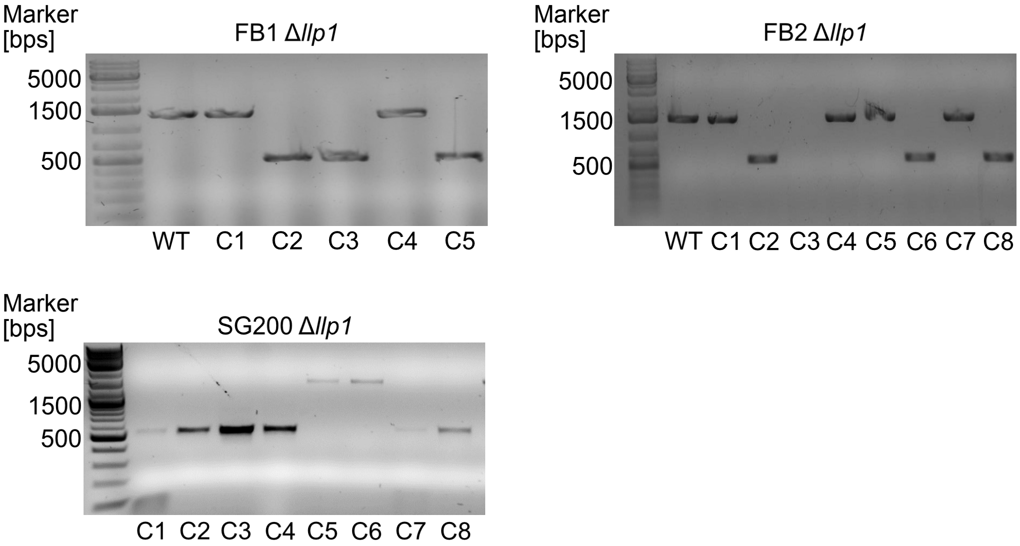

Supplementary Figure S1. Validation of *U. maydis* transformants using colony PCR. Successful gene deletions are confirmed by the presence of 500 bp fragments, whereas unsuccessful deletions, similar to wild-type cells, yield 1500 bp fragments.

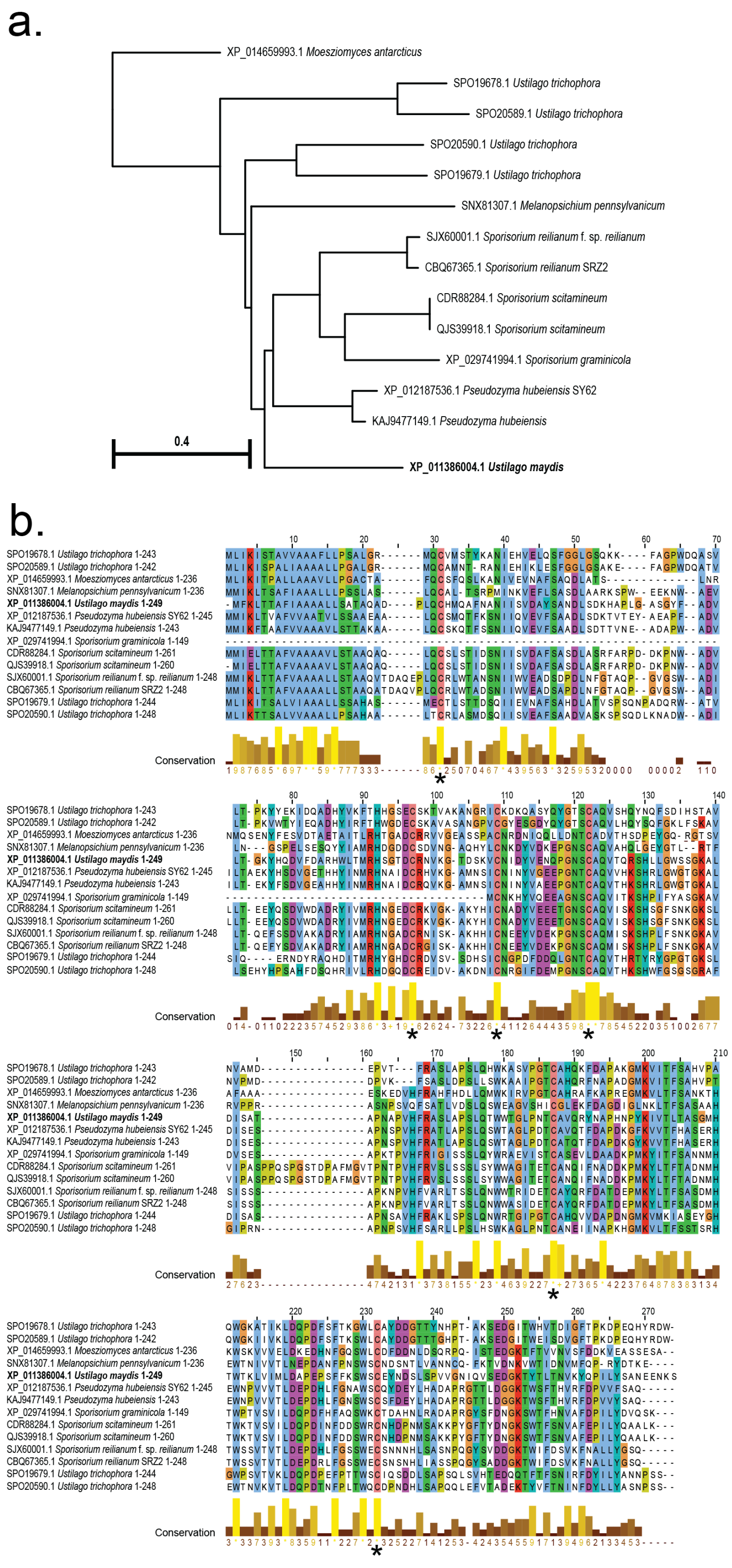

Supplementary Figure S2. Llp1 and homologs are specific to smut fungi. a. All identified orthologues are exclusively found in smut fungi. **b.** Multiple sequence alignment of Llp1 orthologues. Conserved cysteine residues, present across all proteins, are marked with asterisks. Sequence similarities range from 34% to 52%. To improve alignment accuracy, the conservation score excludes the first 108 amino acids of XP_029741994.1 (*Sporisorium graminicola*), the only protein in the dataset without a predicted signal peptide.

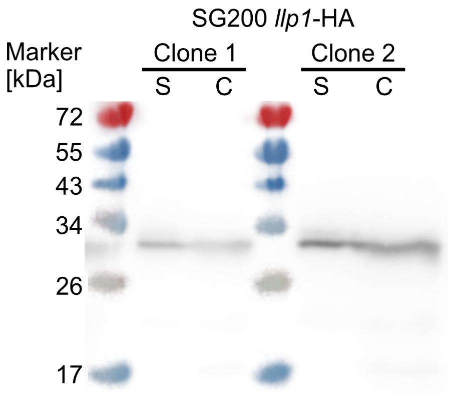

Supplementary Figure S3. Llp1 produced in *U. maydis* associates with cells and is sec reted into the medium. Western blot analysis of TCA-precipitated supernatant samples (S) and pelleted cell fractions (C) from two independent clones expressing C-terminally he magglutinin (HA)-tagged Llp1 integrated at its native locus.

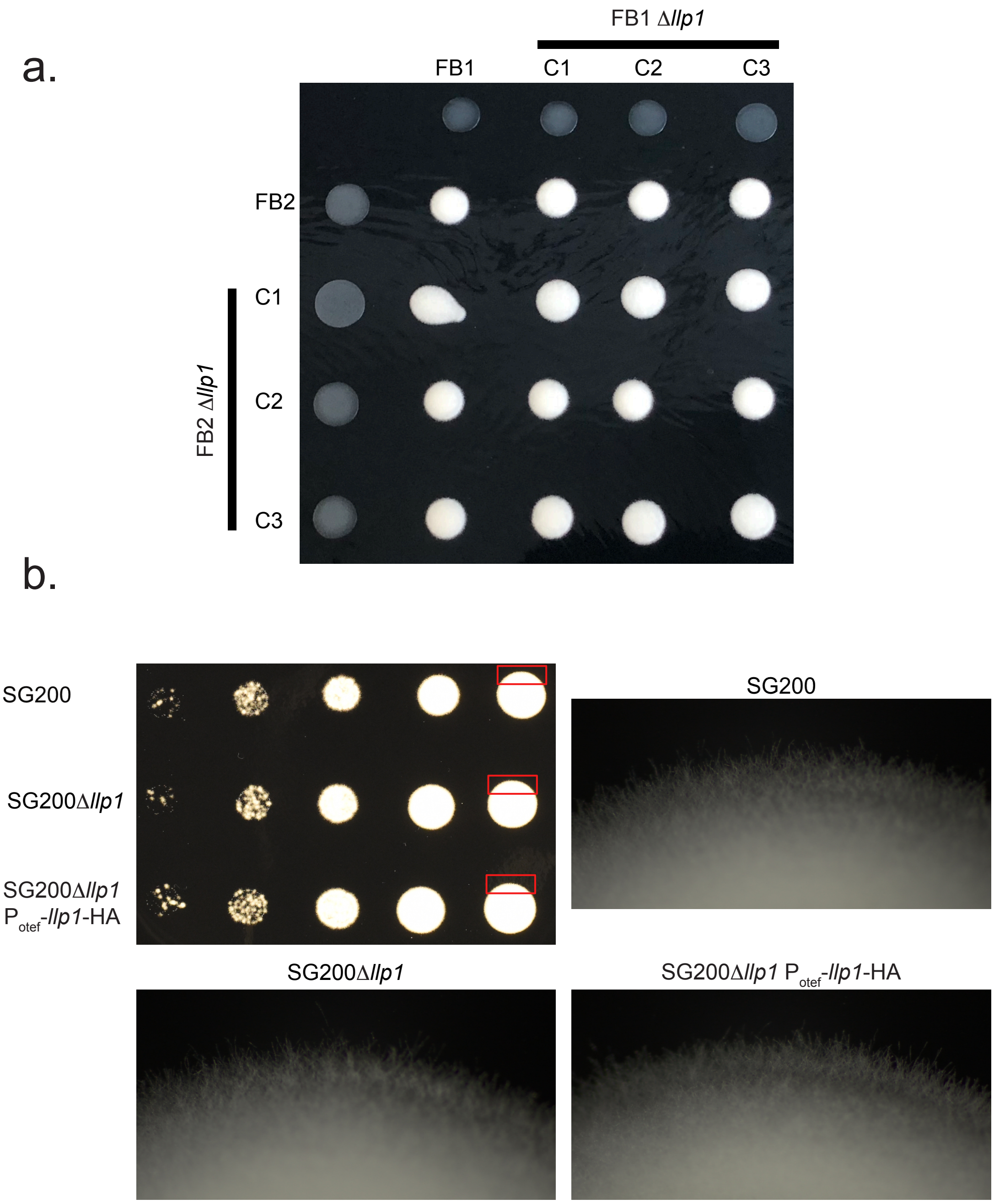

Supplementary Figure S4. Mating - and filamentation assay on PD agar supplemented with charcoal. a: Haploid *U. maydis* strains were spotted individually or mixed in a 1:1 ratio in a grid pattern. White, fuzzie colonies indicate the formation of dikaryotic filaments. **b:** Solopathogenic *U. maydis* SG200 wildtype, a *llp1* knockout strain, and an *llp1* overexpressing strain were spotted in serial dilutions, starting at an OD_{600} of 1. Images were captured using a binocular microscope with 40 × magnification.

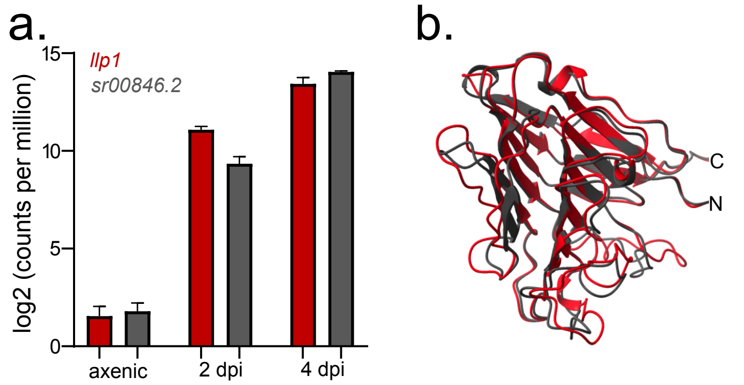

Supplementary Figure S5. The homologue of *UMAG_00027* in *Sporisorium reilianum* (*sr00846.2*) shares a similar transcript regulation pattern and structural similarity. a. Under axenic conditions and during the early stages of infecting the maize plant, the transcripts of both genes exhibit the same regulation pattern. Data is derived from [5] **b.** Structural superposition of the two homologues reveals a similar protein architecture with an RMSD of 4.218 Å across all 243 Cα atoms and a sequence identity of 44.13%.

Supplementary Table S1. Primers used and generated in this s

Number	*Sequence*	*Description*	*Reference*
oMS74-Rv	CGGCGTTCGACTCTT	Reverse primer to generate pMC107	[1]
oFA630	AGGAGGGTCTCCCATGGGCCCTCTGCAATGCCATATGCAG	Forward primer used to generate pMC056	(This study)
oFA631	AGGAGGGTCTCCTCGAGGCTTTTGTTCTCTTCGTTCGC	Reverse primer used to generate pMC056	(This study)
oMC177	CAAAATTCCATTCTACAACGGATCCTCTACTCTGCCAACGGTTTTAGAGC	Forward primer to generate pMC107	(This study)
oMC174	GCTTTTGCTCCTCTTAGCTTTTCAAAATCCATTGCCCGTGATTGCTTGCAGATAGCATCCTAGCAGTTTGCTCACATCTT	Donor DNA for generating *llp1* deletion strains	(This study)
oMC175	AATACCAACCGATCCTCTACTCTGCCAACGAAGAGAACAAGTCTGGATCCTACCCCTACGACGTGCCCGACTATGCCTAAATTGCTTGCAGATAGCATCCTAGCAGTTTGCTCACATCTT	Donor DNA for generating *llp1*-HA strains	(This study)
oAL859	TTAACGTCTCCCATGTTCAAGCTTACTACTGCCTTTATTGCCGCTGCGG	Forward primer used to generate pMC108	(This study)
oAL860	TTAACGTCTCCGGCCGCTTAGGCATAGTCGGGCACGTCGTAGGGGTAAGACTTGTTCTCCTCGTTG	Reverse primer to generate pMC108	(This study)

Supplementary Table S2. Plasmids used and generated in this s

Plasmid	*Usage*	*Citation*
pET24d	Protein overexpression with C-terminal hexahistidine tag	(Novagen)

pMS73	CRISPR/Cas9 vector for mutliplexed genome editing in *Ustilago maydis*. Addgene #110629	[2]
pMC056	Overexpression of Llp1 with a C-terminal hexahistidine tag	(This study)
pMC107	pMS73 with sgRNA to disrupt *UMAG_00027* for gene deletion and C-terminal tagging	(This study)
pMC108	Plasmid contains *llp1-HA* under control of the constitutive otef promoter to be introduced in the *ip* locus of *U. maydis*	(This study)

Supplementary Table S3. *Ustilago maydis* strains used and generated in this s

Strain	*Parental strain*	*Vector*	*Genotype*	*Citation*
SG200	-	-	*a1 mfa2 bW2 bE1*	[3]
FB1	-	-	*a1b1*	[4]
FB2	-	-	*a2b2*	[4]
MC374	SG200	pMC107	*a1 mfa2 bW2 bE1 Δllp1 Clone 3*	(This study)
MC375	SG200	pMC107	*a1 mfa2 bW2 bE1 Δllp1 Clone 8*	(This study)
MC376	SG200	pMC107	*a1 mfa2 bW2 bE1 Δllp1 Clone 12*	(This study)
MC508	SG200	pMC108	*a1 mfa2 bW2 bE1* ip^R [P_{otef}:*llp1-HA*] ip^S	(This study)
MC679	FB1	pMC107	*Δllp1*	(This study)
MC682	FB2	pMC107	*Δllp1*	(This study)

Supplementary Table S4. MST measurements for sugar- and metal-binding inte raction experiments. The experimental setup and the results are detailed in an accompanying Excel file.

Supplementary Tables S5 and S6. Virulence testing of *U. maydis* strains lacking *llp1* were tested in a maize infection assay. Detailed scoring sheets for the infection assays are av ailable in the form of Excel tables.

Supplementary References:

1. Schuster, M.; Schweizer, G.; Reissmann, S.; Kahmann, R. Genome editing in *Ustilago maydis* using the CRISPR-Cas system. *Fungal Genetics and Biology* **2016**, *89*, 3-9, doi:10.1016/j.fgb.2015.09.001.
2. Schuster, M.; Trippel, C.; Happel, P.; Lanver, D.; Reißmann, S. et al. Single and Multiplexed Gene Editing in *Ustilago maydis* Using CRISPR-Cas9. *Bio Protocols* **2018**, *8*, 1-15, doi:10.21769/bioprotoc.2928.
3. Kämper, J.; Kahmann, R.; Bölker, M.; Ma, L.-J.; Brefort, T.; Saville, B.J.; Banuett, F.; Kronstad, J.W.; Gold, S.E.; Müller, O.; et al. Insights from the genome of the biotrophic fungal plant pathogen *Ustilago maydis*. *Nature* **2006**, *444*, 97-101.
4. Banuett, F.; Herskowitz, I. Different a alleles of *Ustilago maydis* are necessary for maintenance of filamentous growth but not for meiosis. *Proceedings of the National Academy of Sciences of the United States of America* **1989**, *86*, 5878-5882.
5. Zuo, W.; Depotter, J.R.L.; Gupta D.K.; Thines, M.; Doehlemann G. Cross-species analysis between the maize smut fungi *Ustilago maydis* and Sporisorium reilianum highlights the role of transcriptional change of effector orthologs for virulence and disease. *New Phytol*. **2021** 232(2):719-733.
